# Impact of clinical characteristics on human chorionic gonadotropin regression after molar pregnancy

**DOI:** 10.6061/clinics/2021/e2830

**Published:** 2021-08-16

**Authors:** Allison A. Gockley, Lawrence H. Lin, Michelle Davis, Alexander Melamed, Anthony Rizzo, Sue Yazaki Sun, Kevin Elias, Donald P. Goldstein, Ross S. Berkowitz, Neil S. Horowitz

**Affiliations:** I Division of Gynecologic Oncology, Department of Obstetrics and Gynecology, Brigham and Women’s Hospital, Boston, USA.; II Department of Obstetrics, Gynecology & Reproductive Biology, Harvard Medical School Boston, MA, USA.; III Department of Pathology, NYU School of Medicine, New York, USA.; IV Division of Gynecologic Oncology, Department of Obstetrics and Gynecology, Massachusetts General Hospital, Massachusetts, USA.; V Centro de Doencas Trofoblasticas, Departamento de Obstetricia e Ginecologia, Hospital das Clinicas HCFMUSP, Faculdade de Medicina, Universidade de Sao Paulo, Sao Paulo, SP, BR.; VI Gynecologic Oncology Program, Susan F. Smith Center for Women’s Cancers, Dana-Farber Cancer Institute/Harvard Cancer Center, Boston, USA.; VII Trophoblastic Tumor Registry, New England Trophoblastic Disease Center, Boston, USA.

**Keywords:** Gestational Trophoblastic Disease, Chorionic Gonadotropin, Molar Pregnancy

## Abstract

**OBJECTIVES:**

This study aimed to determine the effects of age, race/ethnicity, body mass index, and contraception on human chorionic gonadotropin (hCG) regression following the evacuation of a molar pregnancy.

**METHODS:**

This was a retrospective cohort study of 277 patients with molar pregnancies between January 1, 1994 and December 31, 2015. The rate of hCG regression was estimated using mixed-effects linear regression models on daily log-transformed serum hCG levels after evacuation.

**RESULTS:**

There were no differences in hCG half-lives among age (*p*=0.13) or race/ethnicity (*p*=0.16) groups. Women with obesity and hormonal contraceptive use demonstrated faster hCG regression than their counterparts (3.2 *versus.* 3.7 days, *p*=0.02 and 3.4 *versus.* 4.0 days, *p*=0.002, respectively).

**CONCLUSION:**

Age and race/ethnicity were not associated with hCG regression rates. Hormonal contraceptive use and obesity were associated with shorter hCG half-lives, but with unlikely clinical significance. It is important to understand whether the clinical characteristics of patients may influence the hCG regression curve, as it has been proposed as a way to predict the risk of gestational trophoblastic neoplasia.

## INTRODUCTION

Hydatidiform mole is the most common form of gestational trophoblastic disease and is classified as either complete or partial. A complete mole (CM) is classically described as a diploid androgenetic conception with diffuse trophoblastic proliferation, hydropic change, and absent embryonic/fetal tissue, whereas a partial mole (PM) is a triploid pregnancy with embryonic/fetal tissue and associated focal trophoblastic hyperplasia (1).

Following the diagnosis of a molar pregnancy, uterine evacuation is performed, and patients are monitored for progression to gestational trophoblastic neoplasia (GTN) (2). The risk of developing GTN is up to 20% for CM and <5% for PM (3). Human chorionic gonadotropin (hCG) is used as a marker in postmolar monitoring because of the correlation between hCG levels and trophoblastic tumor burden, allowing for early diagnosis and treatment of GTN (2). Abnormal hCG regression patterns have been correlated with the development of GTN (4,5). While abnormal hCG regression is not currently included by the International Federation of Gynecology and Obstetrics (FIGO) or the World Health Organization staging and scoring systems as a criterion in diagnosing GTN, it is used in some countries for diagnosing GTN (6). However, little is known about the potential impact of patient characteristics on hCG regression.

Recent data suggest that specific patient characteristics may affect the course of molar pregnancy and GTN. The risk of GTN following molar pregnancy has been shown to be significantly different between racial and ethnic groups (7,8). Advanced maternal age has also been associated with a higher risk of GTN (9); however, adolescents may have a lower likelihood of progressing from molar pregnancy to GTN (10). Body mass index (BMI) has not affected the risk of progression to GTN or the efficacy of chemotherapy (11); however, whether BMI affects the clinical course of hCG regression curves remains unknown.

The effective use of contraceptives is crucial after evacuation of a hydatidiform mole because a new pregnancy may confound the interpretation of hCG levels (1). Previous studies suggested that hormonal contraception may elevate the risk of GTN (12,13); however, multiple recent studies evaluating lower-dose modern hormonal contraceptives have shown conflicting results (14,15).

The current study was undertaken to further investigate how patient age, race/ethnicity, BMI, and contraception may affect hCG regression following molar evacuation.

## MATERIALS AND METHODS

This was a retrospective cohort study of women registered at the Donald P. Goldstein, MD, Trophoblastic Tumor Registry from the New England Trophoblastic Disease Center (NETDC) from January 1, 1994 to December 31, 2015, with an initial histological diagnosis of a CM or PM. This study was approved by the Partners Healthcare Institutional Review Board (Protocol #2004P001372), and informed consent was waived as all data were deidentified. Patients who were referred for persistently elevated hCG or initially presenting GTN were excluded. The exclusion criteria were as follows: unclassified mole, loss to follow-up before hCG normalization, <2 hCG measurements within 28 days after uterine evacuation, pregnancy during follow-up, received either prophylactic chemotherapy or hysterectomy, or a coexisting normal fetus.

Histological diagnosis and classification of hydatidiform moles were established by specialists in gynecologic pathology at the Brigham and Women’s Hospital, Department of Pathology, Division of Women’s and Perinatal Pathology. Flow cytometry for ploidy and/or p57 staining was performed as necessary to confirm the diagnosis.

All electronic and paper charts were reviewed. After uterine evacuation, hCG was measured weekly until three measurements were <5 mIU/mL and then monthly for 6 months. All used hCG detection kits were based on chemiluminescent methods with a sensitivity of ≥5 mIU/mL. Patients who were ultimately diagnosed with GTN during follow-up were included in the analysis with the hCG values after diagnosis excluded. GTN was diagnosed according to the 2002 International FIGO criteria as follows: rise of ≥10% of hCG levels in 3 weekly values, plateau (<10% variation) in hCG values for 4 weeks, metastatic disease before plateau or rise, and histological diagnosis of choriocarcinoma. After GTN diagnosis, patients were staged according to the FIGO (2002) anatomic and prognostic system.

Age was defined as <20 years, 20-29 years, 30-39 years, and ≥40 years. Race/ethnicity was categorized as white, black, Asian, or Hispanic based on patient self-identification. BMI was categorized as non-obese (<30.0 kg/m^2^) and obese (≥30.0 kg/m^2^). Contraceptives were categorized as hormonal (HC), which included combined hormonal pills, combined hormonal injections, vaginal rings, progesterone-only pills, progesterone implants/intrauterine devices, and progesterone-only injections, or non-hormonal (NHC), which included condoms, spermicidal solutions, diaphragms, sexual abstinence, and non-hormonal intrauterine devices.

Analysis of the regression of serum hCG concentration after molar evacuation utilized serum hCG levels obtained within 28 days of uterine evacuation. For patients who underwent a second uterine evacuation within 28 days of primary evacuation, only hCG values obtained after the latter procedure were analyzed. Only pre-progression hCG values were analyzed in patients who progressed to GTN.

hCG regression was modeled assuming first-order kinetics. The daily percentage change in serum hCG was estimated using a mixed-effects linear regression model with patient-level random effects to account for repeated measures. Natural log-transformed hCG values were modeled as a function of post- uterine evacuation day (16).

Half-life was calculated as *t*_1/2_=In(2)/β, with t_1/2_ as the half-life and β as the regression coefficient per post-evacuation day. To assess whether the rate of hCG kinetics varied by age, race/ethnicity, BMI, and contraceptive type, we constructed separate models including all variables of interest as covariates and used the likelihood ratio test to determine whether including an interaction term between the variable of interest and days since evacuation improved the model. Statistical significance was set at *p*<0.05.

To assess whether the associations between hCG regression rates and variables of interest could be explained by confounding by age, we repeated the likelihood ratio test used in the main analysis, including a term for age and race/ethnicity, when applicable.

## RESULTS

**Figure 1:**
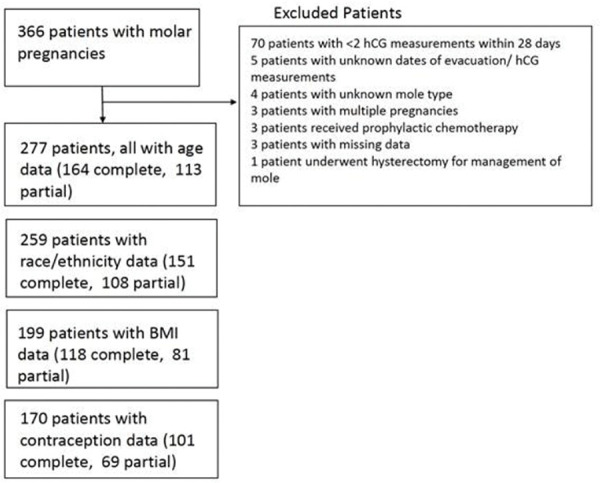
Flowchart of patients with complete or partial molar pregnancies. The chart describes the excluded patients as well as the number of patients with complete and partial mole in each subgroup analysis.

A total of 366 women with an initial diagnosis of hydatidiform moles were observed between January 1994 and December 2015 at the NETDC (Figure 1). Among them, four patients with unclassified molar pregnancy type, three patients with multiple pregnancies with hydatidiform moles and normal fetuses, five patients with unknown date of uterine evacuation, one patient who underwent hysterectomy as treatment for molar pregnancy, three patients who received prophylactic chemotherapy, 70 patients who had <2 hCG measurements within 28 days of molar evacuation, and three patients with missing data were excluded. After exclusions, 277 patients were included in the analysis. All patients had available data on age, 259 had available data on race/ethnicity, 199 had available BMI values, and 170 had available data on contraceptives used. Thirty-four patients were ultimately diagnosed with GTN (31 with stage I disease and three with stage III disease). The clinical characteristics of the patients in this study are described by the type of molar pregnancy in Table 1. Clinical characteristics were similar between complete and partial molar patients, except for age and race. Patients aged 14-19 years had more complete molar pregnancies than partial molar pregnancies (12.2% *versus* 1.8%). Asian women were diagnosed with a complete molar pregnancy more often than with a partial molar pregnancy (14% *versus* 0.9%). Women with complete molar pregnancies presented with a higher median pre-evacuation hCG level than women with partial molar pregnancies (151,815 *versus* 108,418, *p*=0.02).

**Figure S1:**
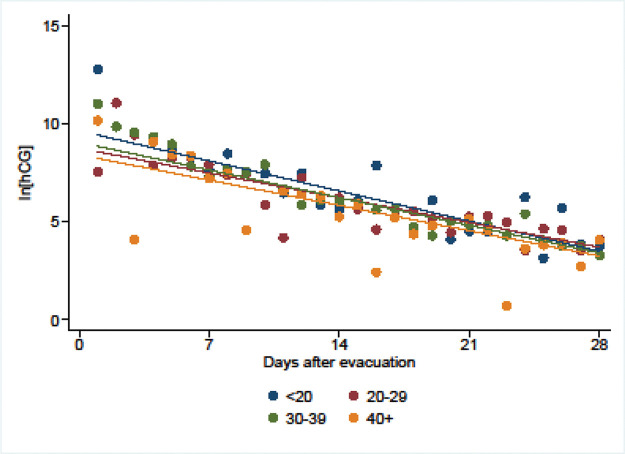
Human chorionic gonadotropin (hCG) regression curves by age for all molar pregnancies. Each point represents the mean hCG level. No significant difference was noted in the rate of hCG regression by age for all (*p*=0.13), complete (*p*=0.18), or partial (*p*=0.83) molar pregnancies.

The clinical characteristics of the patients by age are shown in Table S1. Among the patients, 142 (51.3%) were between 30 and 39 years of age and 164 (59.2%) had a CM. The hCG half-life varied between 3.1 and 4.0 days. There were no significant differences between the hCG half-lives by age, including all molar pregnancies (*p*=0.13), and complete (*p*=0.18) or partial molar pregnancy (*p*=0.83) (Table S2 and Figure S1).

**Figure S2:**
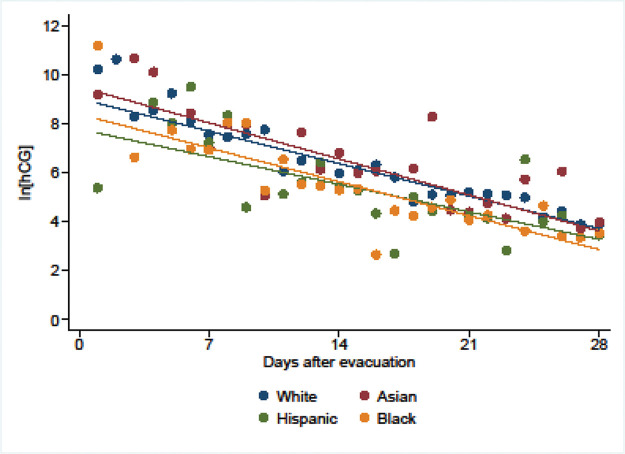
Human gonadotropin (hCG) regression curves by race/ethnicity for all molar pregnancies. Each point represents the mean hCG level. No significant differences were noted in the rate of hCG regression by race for all (*p*=0.16), complete (*p*=0.51), or partial (*p*=0.41) molar pregnancies.

Clinical characteristics according to race/ethnicity are shown in Table S3. Most patients were white (n=173, 66.8%). There were significant variations in the median patient age according to race/ethnicity (white, 32 years; Asian, 31.5 years; Hispanic, 24.5 years; and black, 29 years; *p*=0.001). The type of mole also varied among races/ethnicities, with a higher percentage of CMs in Asians (*p*<0.001). However, the hCG half-life did not vary significantly between race/ethnic groups regardless of mole type (*p*=0.16, *p*=0.51, and *p*=0.41 upon including all, CMs, and PMs, respectively) (Table S4 and Figure S2).

**Figure S3:**
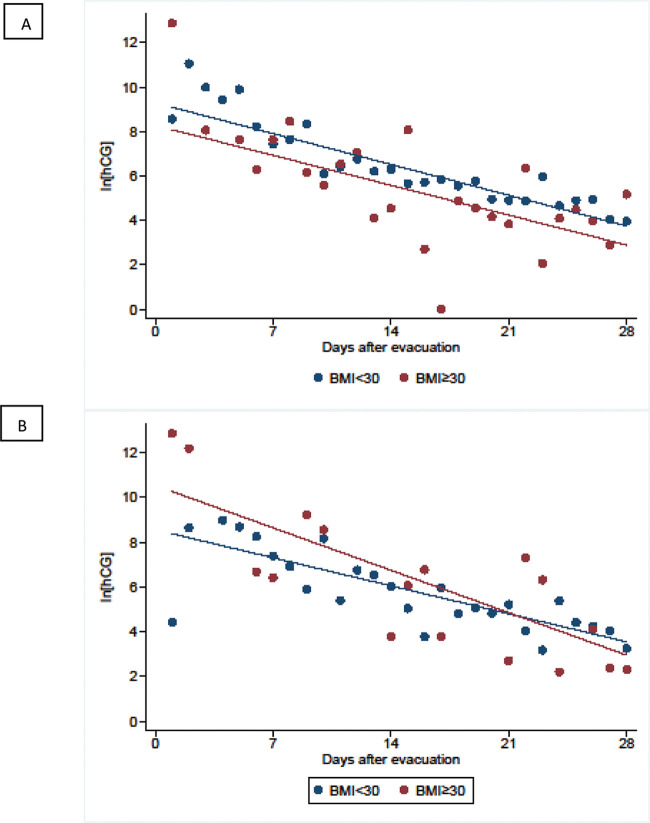
Human gonadotropin (hCG) regression curves by body mass index (BMI) for (A) complete and (B) partial molar pregnancies. Each point represents the mean hCG level. The rate of hCG regression was significantly higher among patients with obesity (BMI≥30 kg/m^2^) than in those without obesity (*p*=0.02). This difference was significant among women with partial molar pregnancies (*p*=0.03), but not among women with complete molar pregnancies (*p*=0.19).

There were 199 patients with available BMI data for analysis. Among them, 171 and 28 were without and with obesity, respectively. There were statistically significant differences in race/ethnicity composition between the obese and non-obese groups (*p*=0.006). Specifically, there was a higher percentage of black and Hispanic women and a lower percentage of Asian women in the obese group (Table S5). Analysis using the mixed-effects linear regression model showed a significant difference in the hCG half-lives between women with and without obesity (*p*=0.02), with women with obesity having a faster rate of hCG regression. This difference persisted for patients with PMs (*p*=0.03) but not for patients with CMs (*p*=0.19). This difference persisted after adjusting for age and race/ethnicity groups (*p*=0.03). However, the absolute difference in regression half-life was less than a single day (3.7 days and 3.2 days for patients with and without obesity, respectively) (Table S6, Figure S3).

Data for contraceptive use was available in 170 patients. Among them, 107 utilized HC (90 used combined estrogen-progesterone methods and 17 used progesterone-only contraception) and 63 utilized NHC.

**Figure S4:**
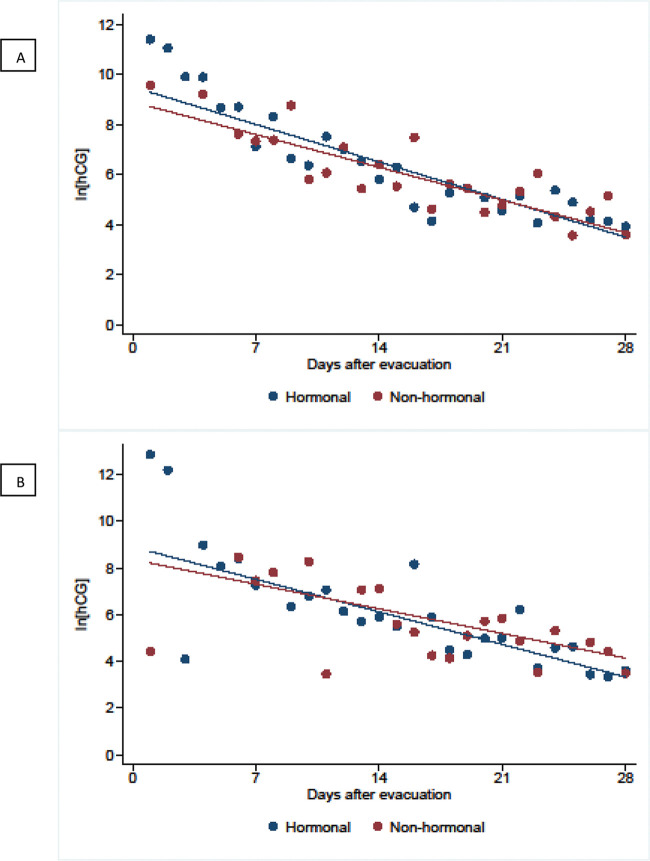
Human gonadotropin (hCG) regression curves by contraception for (A) complete and (B) partial molar pregnancies. Each point represents the mean hCG level. The hCG regression rate was significantly faster in patients using combined hormonal contraception than in those using non-hormonal contraception (*p*=0.002). The difference was significant among patients with partial molar pregnancies (*p*<0.001) but not in those with complete molar pregnancies (*p*=0.32).

The demographic characteristics of patients whose post-evacuation method of contraception was known are tabulated in Table S7. Patients using HC were younger than those using NHC (*p*<0.001)**.** Women using HC had a significantly shorter hCG half-life (3.4 d) than those using NHC (4.0 days, *p*=0.002). This difference was observed among patients with PMs, but not among patients with CMs (Table S8 and Figure S4). The rate of hCG regression differed between the HC and NHC groups even after adjusting for age differences (*p*<0.001).

## DISCUSSION

While the postmolar hCG regression curve has been proposed as a tool for early detection of GTN (4), potential patient-level factors that may influence hCG regression have not been extensively studied. To our knowledge, this is the first study to investigate the potential impact of clinical characteristics on hCG regression kinetics following uterine evacuation of molar pregnancies. In this study, age and race/ethnicity did not affect the hCG regression rates. The hCG half-lives were shorter among women with obesity and HC users. However, these differences were <1 day and not clinically significant. These findings suggest that age, race/ethnicity, BMI, and contraception are unlikely to significantly affect the hCG regression kinetics. Patients can be counseled that their hCG regression rates following molar pregnancy are likely to be similar to classic regression curves, regardless of these factors.

In this study, patients with CMs and PMs were analyzed separately to investigate any differences between the effects of these clinical characteristics on hCG regression kinetics based on the type of molar pregnancy. For age and race/ethnicity, there were no differences in hCG regression among all or each type of mole. In contrast, women with obesity had faster hCG regression rates among all moles and PMs; however, this difference was not significant among patients with CMs. A similar trend was observed in terms of contraceptive use, as women utilizing NHC experienced a longer hCG half-life than women utilizing HC, which was significant for PMs and not for CMs.

One possible explanation for the different hCG regression behavior depending on the mole type is that a CM is more abnormal with pronounced trophoblastic proliferation than a PM. CM pregnancies may be less influenced by the clinical factors investigated in this study. Interestingly, both significant differences in hCG regression observed in this study are potentially linked to hormonal factors. Obesity is associated with higher levels of circulating estrone due to peripheral conversion from androstenedione (17). However, obesity and HC use demonstrated lower circulating levels of estradiol due to ovarian suppression (18,19). Currently, there are conflicting laboratory data on the influence of circulating hormone levels on hCG secretion by trophoblastic cells (20,21).

Data from the 1970s demonstrated an increased risk of postmolar GTN and a longer time to hCG remission among patients receiving higher HC doses (>50 mcg of ethinyl estradiol) (13). Other contemporary studies investigating the association of HC and GTN progression among low-dose HC users did not find any association between HC and progression to GTN or time to hCG remission (14,15).

Another possible explanation for the differences observed among women with obesity and those using HC is the potential changes that these factors may have on renal function. Since hCG is excreted in the urine, any factor that affects renal function may affect hCG excretion and the observed serum regression curve (22). Obesity increases the glomerular filtration rate via a compensatory hyperfiltration mechanism, possibly resulting in rapid hCG clearance (23). HC use has also increased glomerular filtration rates (24).

The differences in the hCG half-life observed were not clinically significant, as there was <1 day difference between the half-life among groups. Patients should be counseled that traditional hCG monitoring is reasonable and that these factors do not necessitate an individualized follow-up plan. Minor variances in the hCG half-life caused by BMI and HC use are not likely to have a clinically significant effect on the duration of the required follow-up in patients following molar evacuation.

Limitations of this study include its retrospective nature and the small number of patients in some subgroups. Additionally, as this data is from a tertiary care referral center, selection bias may limit the generalizability of these findings. Given the rarity of molar pregnancy; however, this study represents the first and largest report on how clinical characteristics may affect hCG regression following molar evacuation. Another strength of this study was that it included data from patients seen in a single institution by the same team, which employed standardized management protocols.

Age, race/ethnicity, BMI, and contraception type do not appear to have a clinically significant impact on hCG regression, as measured in hCG half-lives. While significant differences were observed among women with obesity and those using hormonal contraception, these differences are unlikely to have clinical implications in routine hCG monitoring. Women should be counseled that following molar evacuation, these factors do not meaningfully affect hCG regression and do not diminish the usefulness of hCG regression curves in predicting the risk of postmolar GTN.

## AUTHOR CONTRIBUTIONS

This project was conceived by Gockley AA, Lin LH, Melamed A, Berkowitz RS, Elias K and Horowitz NS. Data collection was completed by Gockley A, Lin LH, Sun SY and Rizzo A. Data analysis was conducted by Melamed A and Rizzo A. Project guidance provided by Berkowitz RS, Horowitz NS, Elias K, Goldstein DP and Davis M. Gockley A and Lin LH wrote the manuscript, which was reviewed by all co-authors.

## APPENDIX Supplementary Material

**Table 1:** Clinical characteristics of patients by the type of molar pregnancy

	Complete mole (n=164)	Partial mole (n=113)	*p*
Age (years), n (%)			0.006*
14-19	20 (12.2)	2 (1.8)	
20-29	51 (31.1)	35 (31.0)	
30-39	76 (46.3)	66 (58.4)	
40-53	17 (10.4)	10 (8.8)	
Race/Ethnicity, n (%)			<0.001†
White	86 (52.4)	85 (75.2)	
Asian	23 (14.0)	1 (0.9)	
Hispanic	22 (13.4)	10 (8.8)	
Black	20 (12.2)	10 (8.8)	
Other/Unknown	13 (7.9)	7 (6.2)	
Contraceptive type, n (%)			0.34†
Hormonal	33 (20.1)	30 (26.5)	
Non-hormonal	69 (42.1)	39 (34.5)	
Unknown	62 (37.8)	44 (38.9)	
Body mass index classification, n (%)			0.64†
Non-obese	99 (60.4)	72 (63.7)	
Obese	19 (11.6)	9 (8.0)	
Unknown	46 (28.0)	32 (28.3)	
Gravidity, median (IQR)	2 (1-3)	2 (2-3)	0.16*
Parity, median (IQR)	0.5 (0-1)	1 (0-1)	0.08*
Pre-evacuation hCG, median (IQR)	151,815 (75,954-322,957)	108,418 (26,536-282,000)	0.02*
History of prior mole, n(%)	7 (4.3)	2 (1.8)	0.21†
hCG values, median (IQR)	3 (2-4)	3 (2-3)	0.96*

* Kruskal-Wallis test

† Fisher’s exact test.

IQR, interquartile range; hCG, human chorionic gonadotropin

**Table S1:** Characteristics of women with hydatidiform molar pregnancies by age.

	<20 (n=22)	20-29 (n=86)	30-39 (n=142)	≥40 (n=27)	*p* value
Race/Ethnicity, n(%)					<0.001†
White	10 (45.5)	41 (47.7)	103 (72.5)	17 (63.0)	
Asian	1 (4.5)	6 (7.0)	14 (9.9)	3 (11.1)	
Hispanic	7 (31.8)	17 (19.8)	5 (3.5)	3 (11.1)	
Black	1 (4.5)	15 (17.4)	10 (7.0)	4 (14.8)	
Other/Unknown	3 (13.6)	7 (8.1)	10 (7.0)	0 (0)	
Gravidity, median (IQR)	1 (1-1)	2 (1-3)	2 (2-3)	3 (2-4)	<0.001*
Parity, median (IQR)	0 (0-0)	0 (0-1)	1 (0-1)	1 (0-2)	<0.001*
Preevacuation hCG, median (IQR)	207,761 (87,736-916,197.5)	136,612 (66,623-136,612	128,853 (43,224-284,240	138,741 (82,042-310,073)	0.25*
Type of Mole, n (%)					0.006†
Complete mole	20 (90.9)	51 (59.3)	76 (53.5)	17 (63.0)	
Partial mole	2 (9.1)	35 (40.7)	66 (46.5)	10 (37.0)	

* Kruskal-Wallis test

† Fisher exact test

IQR - interquartile range, hCG - human chorionic gonadotropin.

**Table S2:** Comparison of hCG half-lives by age group among women with hydatidiform molar pregnancies with 95% Confidence Intervals.

	<20	20-29	30-39	≥40	*p* value^a^
All moles	3.3 (2.9-3.7)	3.6 (3.4-3.8)	3.7 (3.5-3.8)	3.3 (3.0-3.7)	0.13
Complete mole	3.3 (2.9-3.7)	3.5 (3.2-3.8)	3.5 (3.3-3.7)	3.1 (2.8-3.5)	0.18
Partial mole	---	3.8 (3.4-4.2)	4.0 (3.7-4.2)	3.8 (3.2-4.7)	0.83

a Calculated using mixed effects linear regression to account for non-independence of repeated measures.

hCG - human gonadotropin.

**Table S3:** Characteristics of women with hydatidiform molar pregnancies by race/ethnicity.

	White n=171	Asian n=24	Hispanic n=32	Black n=30	*p* value
Age, median (IQR)	32 (29-35)	31.5 (26.5-36)	24.5 (20.5-31.5)	29 (25-35)	0.001*
Gravidity, median (IQR)	2 (1-3)	2 (1.5-3)	2 (1-4)	3 (2-4)	0.01*
Parity, median (IQR)	1 (0-1)	0 (0-1)	1 (0-2)	1 (0-2)	0.05*
Pre-evacuation hCG, median (IQR)	137,875 (665,425-325,445)	226,591 (108,084-345,760)	100,711 (41,655-310,3000	82,399 (26,536-156,082)	0.08*
Type of Mole, n (%)					<0.001†
Complete mole	86 (50.3)	23 (95.8)	22 (68.7)	20 (66.7)	
Partial mole	85 (49.7)	1 (4.2)	10 (31.3)	10 (33.3)	
History of prior mole, n (%)	3 (1.8)	3 (12.5)	1 (3.1)	1 (3.3)	0.05†
hCG measurements	3 (2-4)	3 (2.5-4)	3 (2-3.5)	2.5 (2-3)	0.09*

* Kruskal-Wallis test

† Fisher exact test

IQR - interquartile range, hCG - human chorionic gonadotropin.

**Table S4:** Comparison of hCG half-lives by race/ethnicity among women with hydatidiform molar pregnancies, adjusted for age, with 95% Confidence Intervals.

	All races	White	Asian	Hispanic	Black	*p* value^a^
All moles	3.6 (3.5-3.7)	3.7 (3.5-3.8)	3.2 (2.9-3.7)	3.8 (3.4-4.3)	3.4 (3.0-3.9)	0.16
Complete mole	3.4 (3.3-3.6)	3.5 (3.3-3.7)	3.2 (2.9-3.7)	3.8 (3.3-4.4)	3.3 (2.8-4.1)	0.51
Partial mole	3.9 (3.6-4.1)	3.9 (3.7-4.3)	3.7 (3.5-3.9)	3.8 (3.3-4.6)	3.3 (2.8-4.5)	0.41

a Calculated using mixed effects linear regression to account for non-independence of repeated measures.

hCG - human gonadotropin.

**Table S5:** Characteristics of women with hydatidiform molar pregnancies by body mass index (BMI).

	Non-obese (BMI <30kg/m2) n=171	Obese (BMI ≥30kg/m2) n=28	*p* value
Age, median (IQR)	30 (24-34)	30.5 (27.5-36)	0.28*
Race/Ethnicity, n(%)			0.007†
White	109 (63.7)	13 (46.4)	
Asian	17 (9.9)	1 (3.6)	
Black	14 (8.2)	9 (32.1)	
Hispanic	22 (12.9)	5 (17.9)	
Unknown/Other	9 (5.3)	0 (0)	
Gravidity, median (IQR)	2 (1-3)	2 (1.5-4)	0.30*
Parity, median (IQR)	1 (0-1)	1 (0-2)	0.20*
Pre-evacuation hCG, median (IQR)	138,709 (53,738.5-336,870)	146,054 (54,786-320,000)	0.80*
Type of Mole, n (%)			0.41†
Complete mole	99 (57.9)	19 (67.9)	
Partial mole	72 (42.1)	9 (32.1)	
History of prior mole, n (%)	7 (4.1)	1 (3.6)	1.0†
hCG measurements, median (IQR)	3 (2-4)	3 (2-3.5)	0.42*

* Kruskal-Wallis test

† Fisher exact test

IQR - interquartile range, hCG - human chorionic gonadotropin, BMI - body mass index.

**Table S6:** Comparison of hCG half-lives (in days) by body mass index (BMI) among women with hydatidiform molar pregnancies, adjusted for age and race/ethnicity with 95% Confidence Intervals.

	Non-obese (BMI <30kg/m^2^)	Obese (BMI ≥30kg/m^2^)	*p* value^a^
All moles	3.7 (3.5-3.8)	3.2 (2.9-3.6)	0.02
Complete moles	3.6 (3.4-3.8)	3.2 (2.8-3.7)	0.19
Partial moles	3.9 (3.6-4.2)	3.2 (2.7-3.9)	0.03

a Calculated using mixed effects linear regression to account for non-independence of repeated measures.

hCG - human chorionic gonadotropin.

**Table S7:** Characteristics of women with hydatidiform molar pregnancies by contraception type.

	Hormonal contraception n=107	Non-hormonal contraception n=63	*p* value
Age, median (IQR)	29 (22-34)	32 (30-36)	<0.001*
Race/Ethnicity, n(%)			0.004†
White	58 (56.9)	45 (71.4)	
Asian	6 (5.6)	9 (14.3)	
Black	21 (19.6)	3 (4.8)	
Hispanic	17 (15.9)	5 (7.9)	
Unknown	5 (4.7)	1 (1.6)	
Gravidity, median (IQR)	2 (1-3)	2 (1-3)	0.77*
Parity, median (IQR)	0.5 (0-1)	1 (0-1)	0.52*
Pre-evacuation hCG, median (IQR)	148,088 (51,114-363,450)	133,844 (51,192-327,932)	0.71*
Type of Mole, n (%)			0.20†
Complete mole	68 (63.5)	33 (52.4)	
Partial mole	39 (36.5)	30 (47.6)	
History of prior mole, n (%)	3 (2.8)	2 (3.2)	1.0†

* Kruskal-Wallis test

† Fisher exact test

IQR - interquartile range, hCG - human chorionic gonadotropin.

**Table S8:** Comparison of hCG half-lives (in days) by contraception type among women with hydatidiform molar pregnancies, adjusted for age with 95% Confidence Intervals.

	Hormonal contraception	Non-hormonal contraception	*p* value^a^
All moles	3.4 (3.2-3.6)	4.0 (3.7-4.3)	0.002
Complete moles	3.4 (3.2-3.6)	3.6 (3.3-4.0)	0.32
Partial moles	3.4 (3.2-3.7)	4.7 (4.1-5.5)	<0.001

a Calculated using mixed effects linear regression to account for non-independence of repeated measures.

hCG - human chorionic gonadotropin.

## References

[1] Berkowitz RS, Goldstein DP (2009). Clinical practice. Molar pregnancy. N Engl J Med.

[2] Ngan HY, Kohorn EI, Cole LA, Kurman RJ, Kim SJ, Lurain JR (2012). Trophoblastic disease. Int J Gynaecol Obstet.

[3] Lurain JR (2010). Gestational trophoblastic disease I: epidemiology, pathology, clinical presentation and diagnosis of gestational trophoblastic disease, and management of hydatidiform mole. Am J Obstet Gynecol.

[4] Behtash N, Ghaemmaghami F, Honar H, Riazi K, Nori A, Modares M (2004). Is normal beta-hCG regression curve helpful in the diagnosis of persistent trophoblastic disease?. Int J Gynecol Cancer.

[5] Matsui H, Iitsuka Y, Yamazawa K, Tanaka N, Mitsuhashi A, Seki K (2003). Criteria for initiating chemotherapy in patients after evacuation of hydatidiform mole. Tumor Biol.

[6] Yedema KA, Verheijen RH, Kenemans P, Schijf CP, Borm GF, Segers MF (1993). Identification of patients with persistent trophoblastic disease by means of a normal human chorionic gonadotropin regression curve. Am J Obstet Gynecol.

[7] Gockley AA, Joseph NT, Melamed A, Sun SY, Goodwin B, Bernstein M (2016). Effect of race/ethnicity on clinical presentation and risk of gestational trophoblastic neoplasia in patients with complete and partial molar pregnancy at a tertiary care referral center. Am J Obstet Gynecol.

[8] Maestá I, Berkowitz RS, Goldstein DP, Bernstein MR, Ramirez LA, Horowitz NS (2015). Relationship between race and clinical characteristics, extent of disease, and response to chemotherapy in patients with low-risk gestational trophoblastic neoplasia. Gynecol Oncol.

[9] Elias KM, Shoni M, Bernstein M, Goldstein DP, Berkowitz RS (2012). Complete hydatidiform mole in women aged 40 to 49 years. J Reprod Med.

[10] Braga A, Growdon WB, Bernstein M, Maestá I, Rudge MV, Goldstein DP (2012). Molar pregnancy in adolescents. J Reprod Med.

[11] Maestá I, Horowitz NS, Goldstein DP, Bernstein MR, Ramírez LA, Moulder J (2015). Response to chemotherapy in overweight/obese patients with low-risk gestational trophoblastic neoplasia. Int J Gynecol Cancer.

[12] Stone M, Dent J, Kardana A, Bagshawe K (1976). Relationship of oral contraception to development of trophoblastic tumour after evacuation of a hydatidiform mole. Br J Obstet Gynaecol.

[13] Bagshawe KD, Stone M (1980). Oral contraceptives and post-molar trophoblastic tumours. Lancet.

[14] Braga A, Maestá I, Short D, Savage P, Harvey R, Seckl MJ (2016). Hormonal contraceptive use before hCG remission does not increase the risk of gestational trophoblastic neoplasia following complete hydatidiform mole: a historical database review. BJOG.

[15] Dantas PRS, Maestá I, Filho JR, Junior JA, Elias KM, Horowitz N (2017). Does hormonal contraception during molar pregnancy follow-up influence the risk and clinical aggressiveness of gestational trophoblastic neoplasia after controlling for risk factors?. Gynecol Oncol.

[16] Van Trommel NE, Sweep FC, Ross HA, Massuger LF, Thomas CM (2006). Comparison of human chorionic gonadotropin +beta and invasive trophoblast antigen disappearance rates in serum after evacuation of molar pregnancy. Int J Mol Med.

[17] Kirschner MA, Ertel N, Schneider G (1981). Obesity, hormones, and cancer. Cancer Res.

[18] Legro RS, Pauli JG, Kunselman AR, Meadows JW, Kesner JS, Zaino RJ (2008). Effects of continuous versus cyclical oral contraception: a randomized controlled trial. J Clin Endocrinol Metabol.

[19] Freeman EW, Sammel MD, Lin H, Gracia CR (2010). Obesity and reproductive hormone levels in the transition to menopause. Menopause.

[20] Gal D, Simpson ER, Porter JC, MacDonald PC, Buchsbaum HJ (1981). Failure of contraceptive steroids to modify human chorionic gonadotrophin secretion by hydatidiform mole tissue and choriocarcinoma cells in culture. Steroids.

[21] Shoni M, Nagymanyoki Z, Vitonis AF, Jimenez C, Ng SW, Quade BJ (2013). p-21-Activated kinase-1, -4 and -6 and estrogen receptor expression pattern in normal placenta and gestational trophoblastic diseases. Gynecol Oncol.

[22] Birken S, Kovalevskaya G, O'Connor J (1996). Metabolism of hCG and hLH to multiple urinary forms. Mol Cell Endocrinol.

[23] Atthobari J, Gansevoort RT, Visser ST, de Jong PE, de Jong-van den Berg LT, PREVEND Study Group (2007). The impact of hormonal contraceptives on blood pressure, urinary albumin excretion and glomerular filtration rate. Br J Clin Pharmacol.

[24] Brandle E, Gottwald E, Melzer H, Sieberth HG (1992). Influence of oral contraceptive agents on kidney function and protein metabolism. Eur J Clin Pharmacol.

